# Seroprevalence of Hepatitis E Virus in Forestry Workers from Trentino-Alto Adige Region (Northern Italy)

**DOI:** 10.3390/pathogens9070568

**Published:** 2020-07-14

**Authors:** Marina Monini, Fabio Ostanello, Alessandra Dominicis, Valentina Tagliapietra, Gabriele Vaccari, Annapaola Rizzoli, Claudia M. Trombetta, Emanuele Montomoli, Ilaria Di Bartolo

**Affiliations:** 1 Department of Food Safety, Nutrition and Veterinary Public Health, Istituto Superiore di Sanità, Viale Regina Elena 299, 00161 Rome, Italy; alessandra.d.k@gmail.com (A.D.); gabriele.vaccari@iss.it (G.V.); ilaria.dibartolo@iss.it (I.D.B.); 2 Department of Veterinary Medical Sciences, University of Bologna, Via Tolara di Sopra, 50, 40064 Ozzano dell’Emilia (BO), Italy; fabio.ostanello@unibo.it; 3 Department of Biodiversity and Molecular Ecology, Research and Innovation Centre, Fondazione Edmund Mach, Via E. Mach, 1, 38098 San Michele all’Adige (TN), Italy; valentina.tagliapietra@fmach.it (V.T.); annapaola.rizzoli@fmach.it (A.R.); 4 Department of Molecular and Developmental Medicine, University of Siena, Via Aldo Moro 2, 53100 Siena, Italy; trombetta@unisi.it (C.M.T.); emanuele.montomoli@unisi.it (E.M.); 5 VisMederi S.r.l., Strada del Petriccio e Belriguardo, 35, 53100 Siena, Italy

**Keywords:** hepatitis E virus, anti-HEV IgG seroprevalence, forestry workers, occupational risk

## Abstract

People with some occupational or recreational activities, such as hunters and veterinarians, may have increased risk to be infected by the hepatitis E virus (HEV). The aim of the present study was to establish whether forestry workers could be considered at a higher risk of HEV infection than a control group. One hundred and fifty sera from forestry workers and a control group of 85 sera were analysed by anti-HEV IgG antibodies detection using a commercial ELISA kit. The anti-HEV IgG seroprevalence was 14% for forestry workers and 9.4% for the control group. Comparing the risk of HEV infection in the two groups, there was no difference in the odds ratio. However, the seroprevalence in older subjects was higher in the forestry workers than in the control group. Two sera from forestry workers were also positive for anti-HEV IgM, and, in one of them, HEV-RNA was detected. Our findings showed an increase of seroprevalence with age, which is likely to reflect cumulative exposure to HEV over time. The occupation of forestry workers did not seem to be associated with a higher risk of HEV infection. The study provided new insights into the risk of acquiring HEV in occupational exposure workers with open-air activities.

## 1. Introduction

Hepatitis E is an acute viral disease caused by the hepatitis E virus (HEV), transmitted by the faecal-oral route [1]. Generally, the infection is self-limiting, but it can become chronic in immunosuppressed patients [1]. HEV is a quasi-enveloped [2], positive-stranded RNA virus coding for three open reading frames (ORF1–3). The virus is classified in the family Hepeviridae. This family has been divided into the genera—Piscihepevirus and Orthohepevirus [3]. The genus *Orthohepevirus* is divided into four species (A–D) [4].

Strains affecting humans belong to five genotypes of Orthohepevirus A: HEV-1–4 and HEV-7. Genotypes HEV-1 and HEV-2 are restricted to humans; genotypes HEV-3 and HEV-4 circulate in developed countries and are zoonotic, with pigs and wild boar as the main reservoirs [5]. Genotype HEV-7 has been detected in dromedary camels and one human case [6,7].

In Europe, the disease is linked to HEV-1 and HEV-2 for subjects travelling in low-income countries, and, in the last years, an increasing number of autochthonous cases due to HEV-3 and HEV-4 have been described. The main route of HEV-3 and HEV-4 transmission is foodborne by the consumption of raw or undercooked pork and wild boar meat [8,9]. Several studies have also suggested that working exposures to pigs or wild boar may represent a risk to be infected by HEV. A higher HEV IgG seroprevalence has been reported in veterinarians working with swine (9.6%) [10], pig farmers (14.1%) [11], abattoir workers (28.3%) [12], hunters (22.2%) [13] and forestry workers (18.0%) [14].

In Italy, the consumption of raw or undercooked wild boar meat and pork products containing the liver has been associated with an increased risk of HEV infection [15]. Furthermore, HEV seropositivity has been significantly associated with occupational exposure to pigs (12.3%) [11].

In Italy, the anti-HEV IgG antibodies seroprevalence in blood donors vary between 1% and 10%, and, in Trentino-Alto Adige region, an area in Northern Italy with a mostly mountainous territory, it is 4.5% [16].

In the Italian wild boar populations, HEV infection is common, and the mean seroprevalence ranges from 4.9% [17] to 56.2% [18]. The infection in other wild animal species has been rarely investigated, but two studies conducted on red deer have confirmed the circulation of HEV-3 with seroprevalence values ranging from 1.2% [19] to 13.9% [20], and to a lesser extent (0.8%) also in chamois [19]. In the present case-control study, the seroprevalence of anti-IgG HEV antibodies in forestry workers (exposed group), in contact with wild animals and in a control group, was investigated, in order to evaluate the potential risk linked to professional activities. Positives anti-IgG sera were also analysed for the presence of anti-IgM antibodies and viral RNA.

## 2. Methods

### 2.1. Study Groups

One-hundred and fifty serum samples from forestry workers were provided by "Fondazione Edmund Mach”, Research and Innovation Centre, Department of Biodiversity and Molecular Ecology (Trento, Italy). The samples were collected from May 2014 to January 2015 from 150 healthy males working for the local forestry service in the Autonomous Province of Trento, the Italian Alps, Northern Italy (6207 km^2^, 541,400 inhabitants, mean altitude: 700 meters above sea level (a.s.l.) and were part of routine health checks carried out by the Provincial Health Authority [21]. The mean age was 48 years (standard deviation SD: 7), and the median age was 49.

The study was planned for independent cases and controls with 0.6 controls per case. The true probability of exposure in forestry workers was assumed to be 25%. To reject the null hypothesis that the exposure rates for case and controls were equal with probability (power) 80%, at least 150 cases and 85 controls should have been tested. The type I error probability associated with this test of this null hypothesis was 5%.

As a control, the second group of 85 serum samples collected in 2013–2014 was obtained from Toscana region by the serum bank of the Laboratory of Molecular Epidemiology, Department of Molecular and Developmental Medicine, University of Siena (Central Italy). Samples were chosen in order to reflect the age and sex composition of the forestry workers group: males with a mean age of 48 years (SD: 14) and the median age of 49. Due to the non-normal distribution of age, the comparison between the 2 groups was performed using the Mann–Whitney U test. The results (U = 6236; *p* = 0.781) indicated that there was no statistically significant difference. Concerning the sex of control and exposed groups, both consisted of males.

Sera were coded and anonymously collected in compliance with European data protection regulation. The only information available was age, sex and year of sampling.

### 2.2. Seroprevalence of IgG Anti-HEV

For seroprevalence estimation, the commercial ELISA test by Wantai (Wantai Biological Pharmacy Enterprise Co., Ltd, Beijing, China) was used. This assay shows specificity and sensitivity for the HEV IgG of 97.96% and 99.6%, respectively [22,23], and was used according to the manufacturer’s instructions. 

### 2.3. Detection of Anti-HEV IgM Antibodies by Western Blotting

Due to budget constraints, as suggested by Spada in previous work [16], the anti-HEV IgG prevalence of the study group being less than 15%, only the anti-HEV IgG positive sera were further analysed for the presence of anti-HEV IgM by Western blotting (WB). The capsid protein derived from genotype 3 swine HEV expressed by a recombinant baculovirus in Sf9 insect cells Δ111Orf2C59 was used as antigen in WB [24].

Transferred protein was incubated with serum samples (1:100), then membranes were stained with anti-human secondary antibodies, anti-IgM (µ-chain specific) conjugated with alkaline phosphatase (1:5000) (Sigma, Saint Louis, MO, US). A serum derived from a naturally infected swine [25] was used as positive control and subsequently detected with an anti-pig IgG (Sigma-Aldrich Co. St. Louis, MO, USA), conjugated with alkaline phosphatase for staining.

### 2.4. Viral Extraction and Real-Time RT-PCR 

Viral RNA was extracted from 100 µL of anti-IgM positive sera using the QIAamp Viral RNA Mini Kit (Qiagen, Redwood City, CA, USA) following manufacturers’ instructions. HEV genome detection was performed using RNA Ultrasense One-Step Quantitative RT-PCR System (Life Technologies, Carlsbad, CA, USA) [26].

### 2.5. Statistical Analysis

Forestry workers were considered as a professionally exposed group. Group values were compared using the χ^2^ test. A *p*-value ≤ 0.05 was considered statistically significant for the analyses. A multivariable logistic regression model was carried out to determine which variables were associated with HEV seroprevalence: age (dichotomised on the median age value, ≤ 48 versus ≥49 years), work category (forestry workers versus control group) and their interaction. The model was based on the simultaneous entry of all variables, and its efficacy was assessed based on the likelihood ratio and the Hosmer–Lemeshow statistic.

The odds ratio (OR) and 95% confidence intervals (95%CI) were calculated from the final multivariable logistic regression model. 

All statistical analyses were performed using the software SPSS 25.0 (IBM SPSS Statistics, New York, NY, USA).

## 3. Results 

Overall, 29/235 tested sera (12.3%; 95%CI: 8.4–17.2) were positive for anti-HEV IgG. The anti-HEV IgG seroprevalence was 14% (21/150, 95%CI: 8.9–20.6) for forestry workers and 9.4% (8/85, 95%CI: 4.2–17.7) for the control group (Table 1). The difference observed in the two groups was not statistically significant. 

Comparing the overall seroprevalence according to the median age, results showed that it increased with age, being higher in subjects ≥ 49 years (17/119, 14.3%; 95%CI: 8.6–21.9) than in subjects ≤ 48 years (12/116, 10.3%; 95%CI: 5.5–17.4). Comparing the seroprevalence within the two study groups, the prevalence in younger subjects (ages below the median) was higher in the control group (6/45, 13.3%; 95%CI: 5.1–26.8) than in forestry workers (6/71, 8.5%; 95%CI: 3.2–17.5; *p* = 0.53). Conversely, seroprevalence in the older subjects (with ages above the median) was higher in the forestry workers (15/79, 19.0%; 95%CI: 11.0–29.4; *p* = 0.04) than in the control group (2/40, 5.0%; 95%CI: 0.6–16.9), and the difference was statistically significant (χ^2^ = 4.24; *p* = 0.04) (Table 1). 

Multivariable logistic regression analysis was performed to evaluate possible associations between the presence of anti-HEV antibodies, professional exposure, median age and considering both median age and professional exposure (Table 2). 

No significant difference in odds ratio (OR) related to compared groups (OR = 1.67; 95%CI: 0.50–5.53) or median age (OR = 2.92; 95%CI: 0.55–15.40) was detected. However, in the multivariable logistic regression, there was the interaction between median age and group. The OR of HEV seroprevalence was significantly (*p* = 0.04) higher in old forestry workers (OR = 7.42; 95%CI: 1.06–51.81) (Table 2). 

The twenty-nine sera, tested positive by ELISA, were further analysed by WB for anti-HEV IgM antibodies detection. Two sera gave a positive result, and they both belonged to the forestry workers’ group (2/235, 0.85%; 95%CI: 0.10–3.04).

Sera positive for both IgG and IgM were also analysed for the presence of viral RNA by One-Step Quantitative Real-Time RT-PCR System. One serum from a forestry worker resulted positive with a mean threshold cycle (Ct) in Real-time value of 36.6. However, no sequences were obtained from the sample.

## 4. Discussion

Studies of seroprevalence of anti-HEV IgG antibodies, conducted in general population and control groups in Europe, have revealed a significant variability among different geographical areas, ranging from 1.3% (Italy) to 52.5% (France) [27] and also varying within the country. The variability may be linked to the different methods used, with a wide range of ELISA tests, which differ for the antigens used, for sensitivity and specificity [28]. Furthermore, the anti IgG seroprevalence rate may be affected by the local eating habits of a particular region [15,16]. For these reasons, comparing seroprevalence data with the results of different studies is difficult to achieve. 

Besides foodborne, which is considered the main route of HEV infections in Europe, other proposed transmission routes are linked to professional exposures of subjects that are in strict and daily contact with the zoonotic HEV reservoirs, such as pigs and other wildlife species (mainly wild boar). In Italy, as well as in other European countries, the human cases are mainly due to HEV-3, which is widespread in domestic pigs (prevalence 26.5–87%; up to 95% in adult pigs) [11,25] and wild boar (prevalence up to 52.2%) [27]. In some areas of the country, the density of wild animals is managed by hunting programs, which involve hunters and forestry workers. In particular, working duties and amount of time spent in the forest make contact probabilities of this latter group with wildlife fauna and their habitat higher.

In the present study, HEV exposure in a group of forestry workers was evaluated by IgG anti-HEV detection in sera. The 14% seroprevalence observed in forestry workers was comparable to values reported in other studies on people with occupational contacts with animals or environmental sources: 18% and 31% in forestry workers in Germany [14] and France [29], respectively, 13% in Norway veterinaries [30], 22.2% [9] and 3.8% [31] in Polish hunters and 12.3% in Italian swine workers [11]. In Italy, previous data on blood donors have reported seroprevalence rates ranging from 8.7% to 9.8% [10,16,32], in line with the results obtained in our control group (10%). 

The seroprevalence in forestry workers was 14%, which was higher than 9.4% in the control group, and the difference was not supported by statistical analyses. This result could be biased by the limited number of our samples and by the different geographical origin of the two groups (northern versus central Italy). The seroprevalence in the two regions has been investigated in a national survey on blood donor [16]. The mean seroprevalence of anti-HEV in males of Trentino-Alto Adige (the region of forestry in this study) was 4.7%, while in the same group in Tuscany (the region of controls in this study), it was 7.9%. Besides, the relative low difference in the mean seroprevalence between the two regions, these values and in particular the value of seroprevalence in Tuscany is near the mean HEV IgG seroprevalence reported in Italy (8.7%) [16].

The importance of confirming the higher risk linked to professional exposure deserves further analyses with more samples. 

One parameter associated with the higher seroprevalence is the age, as resulted by multivariable logistic regression analyses. The odds ratio of HEV seropositivity was significantly higher in older forestry workers (age ≥ 49). The increasing prevalence of anti-HEV IgG with age could be due to exposure to HEV over a longer span of time [33]. Unfortunately, no information is available regarding the number of years on the job as foresters of the subjects involved, but we can suppose that older subjects have been working for a long time than younger. Our findings were in agreement with previous studies, showing an increasing prevalence with age, which is likely to reflect cumulative exposure to HEV over time [29,34] and is therefore considered a predictor of HEV IgG seropositivity [35]. 

On testing the sera positive in ELISA by WB, two of them were found to be positive for IgM, which corresponded to 0.8% of seropositive. Our results agreed with precedent anti-HEV IgM seroprevalence studies conducted in blood donors in Italy [16] and in France [36], indicating an anti-HEV IgM prevalence of 0.4% [16] and 1%, respectively.

The risk factor of being exposed to HEV for forestry workers could be linked not only to their duties but also to their workplace. Contact with wild animals, not only their organs [18] but also with stools spread in the environment [37], may be the sources of HEV infection. 

Another risk factor could be the improper use or wear of personal protective equipment, which, as demonstrated for wild boar hunters, represents a barrier to reduce the exposures to HEV while handling infected animals [38]. Overall, further studies are needed to evaluate the risk for workers and suggest preventive measures. Even if this study has some limitations, such as the number of samples and the different geographical origin of the two analysed groups, it confirms previous findings of the risk of acquiring HEV infection through occupational exposure and, in particular, the role of time spent in a natural environment where wildlife is present. This kind of risk is yet underestimated, especially in Italy, and therefore deserves more attention and further investigations on both human and animal sides.

## Figures and Tables

**Table 1:** Anti-HEV (hepatitis E virus) IgG serum antibodies in forestry workers and control group by median age.

Median Age	Group	ELISA Anti-IgG Positive/Total	Prevalence (%)	95%CI	*p*
≤48	Forestry workers	6/71	8.5	3.2–17.5	0.53
	Control group	6/45	13.3	5.1–26.8	
	Total	12/116	10.3	5.5–17.4	
≥49	Forestry workers	15/79	19.0	11.0–29.4	0.04
	Control group	2/40	5.0	0.6–16.9	
	Total	17/119	14.3	8.6–21.9	
Total	Forestry workers	21/150	14.0	8.9–20.6	0.30
	Control group	8/85	9.4	4.2–17.7	
	Total	29/235	12.3	8.4–17.2	

**Table 2:** Multiple-logistic-regression-derived relative risk (RR) and 95% confidence interval (CI) for anti-HEV IgG in forestry workers vs control group, adjusted for median age and both variables considered together.

Risk Factor	Odds Ratio	95%CI	*p*
Group			
Forestry workers	1.67	0.50–5.53	0.40
Control group	referent	-	-
Age			
≥49	2.92	0.55–15.40	0.21
≤48	referent	-	-
Interaction group × age	7.42	1.06–51.81	0.04
Constant	6.50		0
